# Sun-Protection Habits of Primary Students in a Coastal Area of Greece

**DOI:** 10.1155/2012/629652

**Published:** 2012-09-30

**Authors:** M. Saridi, A. Toska, M. Rekleiti, G. Wozniak, A. Liachopoulou, A. Kalokairinou, K. Souliotis, K. Birbas

**Affiliations:** ^1^General Hospital of Korinthos, 33 Sina Street, 20100 Corinth, Greece; ^2^School of Medicine, University of Thessaly, 41222 Larissa, Greece; ^3^Faculty of Nursing, National and Kapodistrian, University of Athens, 11527 Athens, Greece; ^4^Faculty of Social Sciences, University of Peloponnese, 20100 Corinth, Greece

## Abstract

*Aim*. The aim of the present study was to record habits and attitudes of primary school students in Greece regarding sun-protection measures. *Materials and Methods*. 2,163 students with an average age of 9.9 (±1.1) years, studying in 14 schools of a Greek region, constituted our sample. The SPSS 17.0 software was used for the statistical analysis and significance level was set to *P* ≤ 0.05. *Results*. Our sample had an equal gender distribution. 16% of the students belonged to the high-risk group, 70.2% of the participants lived 0–5 km away from the sea (urban area), 84.2% of the students were Greek, and 15.8% had non-Greek nationality. Half of the participants said they wear a hat when under the sun and 72% of them said they use sunscreen. 33.1% of the students said they had a sunburn last summer. Greek students as well as those who lived near the sea had better behaviour patterns regarding sun protection. Finally, children who did not use a sunscreen systematically had suffered sunburns more often than the rest. *Conclusions*. Health education programmes are necessary for students and parents/teachers alike, in order to raise awareness about everyday sun protection.

## 1. Introduction

Harmful effects of UVR solar radiation occupy a good part of the international literature [1]. Today, climatic conditions can allow ultraviolet radiation (UVA, UVB, UVC) to affect skin and eyes and consequently to increase the risk for serious health conditions like skin cancer. UVR solar radiation levels are influenced by a number of factors like sun elevation, latitude close to the equator, high altitude, ozone layer depletion, and ground reflection. Increases in surface UVR irradiance in the 1990s have been observed from spectral measurements at a few stations in the northern hemisphere. This is evident, for example, in the updated record of Greece and other countries from Europe like Germany and The Netherlands [2, 3]. Increases in UVR solar radiation observed during the last two decades in Germany and Greece cannot be explained by changes in ozone amounts alone, and thus it is necessary to include diminishing influences from other factors [4–6].

As the ozone layer becomes depleted, the protective filter provided by the atmosphere is progressively reduced. Consequently, human beings and the environment are exposed to higher UVR levels, in particular higher UVB levels. Small amounts of UVR radiation are beneficial to health and play an essential role in the production of vitamin D, it may also lower the risk of getting some kinds of cancers such ascoloncancer and it is used in the treatment of skin conditions such as psoriasis. However, excessive exposure to UVR radiation is associated with different types of skin damages. Sunburn is a common acute effect, particularly in those with fair skin type and the major environmental risk factor for skin cancer. The carcinogenic and immunosuppressive effects of chronic UVR exposure induce changes that result in cancers—basal cell carcinoma (BCC), squamous cell carcinoma (SCC), and melanoma. Cortical cataract, pinguecula, and pterygium are the most common eye conditions related to sun exposure [7–9]. 

The alarming fact is that melanoma cases, perhaps the most aggressive type of cancer, are increasing and so are other skin cancer types at increasingly younger ages [8, 9]. The incidence of melanoma increased over the course of a decade from 16.38/100,000 in 1995 to 21.25/100,000 in 2005 [10]. There is generally an increase in incidence of melanoma with decreasing latitude. This has been shown within the Nordic countries, the USA, and Australia. However, this relationship does not persist across nonhomogeneous populations-mortality from melanoma is four to six times higher in Nordic countries than in the Mediterranean countries and there is an opposite relationship between melanoma incidence and latitude in Italy [11]. In Greece for 2002, it was estimated that there were 250 deaths due to CMM, 50 because of SCC, and 19 because of BCC [12]. The economic burden of melanoma translates into costs of screening, diagnostic procedures, adjuvant, curative, palliative and end-of-life treatment, medical followups to the doctor's office, and noninvasive/invasive procedures. Equally significant are the costs associated with years of life lost and loss of productivity [13]. Thus, prevention and adoption of sun-protection behaviours from early life is the only way to promote health. Recent studies point out that excessive exposure to the sun during childhood and adolescence, accumulation of solar radiation over the years, numerous episodes of severe sunburns, large number of moles, fair complexion with blond or red hair and light-coloured eyes, living in countries close to the equator, where the sun's rays are more direct, and family history of melanoma are some important risk factors for developing various problems and mainly skin cancer. Children are at a higher risk of suffering damage from exposure to UVR than adults, in particular because their skin is thinner and more sensitive, and even a short time outdoors in the midday sun can result in serious burns, they have a less developed pigmentation system, it does not have a self-defence system and they probably have a long followup over the remaining years of their life to develop skin cancer [14, 15].

Knowledge levels, attitudes, and behaviour patterns of children and teenagers (a high-risk group for melanoma because of increased exposure to the sun) have already been discussed by various international studies [16–23]. There is some evidence that the annual received UV-dose is independent of age and remains constant throughout life [18, 22, 23]. Children spend an estimated 2.5–3.0 hours outdoors each day and may receive three times more UVB rays annually than adults, because they have a greater opportunity for midday sun exposure during the summer months [20, 23]. Conversely, other studies have found that children receive the same amount of ultraviolet doses as adults do, because the recent technological revolution of the 1990s and the advent of various electronic games and computers gave children and adolescents more incentive to stay indoors during the day [1, 15, 22]. In an attempt to prevent further deterioration, some countries with high melanoma incidence rates have launched targeted studies and educational programmes. The majority of those studies have shown that the higher the knowledge levels, the better the attitudes and behaviours [16–21]. Although these programs have been partially successful in improving knowledge related to sun exposure and skin cancer, they have failed to elicit behavioural changes [23].

The 8–12 age group is considered to gain knowledge easily with the right programmes, but it is only natural that family, school, the media, and state policies in general have to play a role too in that direction [23, 24].

In Greece, there have been few studies on this particular subject. In fact, there are no data in the Greek literature regarding knowledge levels and attitudes of this particular age group (8–12 years) about solar radiation, possible risks, and protection measures. Thus, it is necessary to see the real picture, if a comprehensive health education programme is to be planned in the future [25, 26]. Recording students' attitudes and behaviours may help to define and document all needs and deficits, in order to design and implement a targeted intervention programme [25].

The aim of the present study was to investigate and record attitudes and behaviours of primary students regarding solar radiation as well as sun-protection measures.

## 2. Materials and Methods

2,163 primary school students, aged 8–12 years, from one Greek prefecture, made up the study population. The research protocol was first approved by the Pedagogical Institute of the Greek Ministry of Education. Then, the local Director of Primary Education approved the protocol, and finally all head teachers were fully informed. The protocol was also approved by 38 out of 54 local schools. All schools in the prefecture asked to participate but 16 of them did not accept the participation. Twelve schools were excluded since the headmasters did not grant permission for the implementation of the study; eight more schools with only two or three teachers were excluded since the total number of pupils in the three senior grades was under sixty persons per school. Finally, four schools where almost half the parents did not grant consent had also to be excluded. In this study, only 3rd, 4th, and 5th grade pupils were included. 

The students' parents were asked to give signed consent in order for their children to participate in the study. The final study sample included 14 schools in urban and semiurban areas with a total of *n* = 2,680 students. The students' response rate was 80.7%, which, according to the literature, is satisfactory. The study took place from October 2009 to April 2010. This study is part of a doctoral dissertation. Students were given no information about sun protection before they were handed the questionnaires. The intervention programme began just after the questionnaire stage (May 2010-June 2010), and the followup study took place a year after the intervention programme was completed (September 2010–May 2011).

### 2.1. Study Instrument (Questionnaire)

An anonymous questionnaire was used, which was completed by the students themselves; nevertheless, the researcher was always present if the students needed any help. The questionnaire drew from the WHO project “Intersun,” and was based on special programmes of the Australian Ministry of Health (Sun Smart) and the Cancer Research UK—Sun Smart [27–30]. The questionnaire consists of 21 multiple-choice questions apart from the demographics section which consists of closed questions regarding sex, age, nationality, and place of residence. In the main section of the questionnaire, individual characteristics were recorded regarding complexion, eye colour, hair colour, and the existence of freckles and moles on the students' bodies. Then, we recorded the students' knowledge about during which hours should someone avoid sun exposure, or about correct sunscreen use and damages that frequent sun exposure may induce. The final section of the questionnaire examines the students' behaviours regarding sun-protection measures on an everyday basis and especially during the summer. All questions in this section are closed ended, including yes/no (dichotomous) questions and multiple-choice ones.

The questions, apart from the demographics, were aimed at the assessment of the students' attitudes and behaviours regarding sun-protection measures. All of the questions that were chosen for this study are also included in the previously mentioned programmes.

### 2.2. Instrument Standardization-Reliability-Validity

After the questionnaire had been compiled, it was given to 50 students (8–12 years old) for a pilot study and standardization. After minor adjustments, it was given to another 50 students and no problems were detected concerning the completion and the results. The students who took part in the pilot studies were excluded from the final study.

Internal consistency reliability was assessed by Cronbach's alpha, which was 0.70. Face validity of the questionnaire was also assessed (*rs* = 0.78).

### 2.3. Restrictions of the Study

Accessibility to schools (given that they were located to different parts of the region) was initially a major restriction of the present study.

It is also noteworthy that, during the first stage of the study which included questionnaire completion and an intervention programme (October–April 2010), H1NI1 influenza had infected many people in Greece. According to the Greek CDC and Ministry of Health guidelines, classes should not be crowded, so every school grade took part in the study separately and, consequently, the process took more time to complete. 

Finally, an important component of our sample comprised of foreign students who quite often had difficulties in understanding and completing the questionnaire; as a result, the researcher had to assist those students and helped them by explaining all the questions that they had, and that meant that the whole process took more time for each school grade. Those difficulties did not create substantial problems to the study and did not skew the results. The only consequence was that the costs for the researcher were higher and also that the study took more time than expected. 

### 2.4. Statistical Analysis

Mean values, standard deviation, medians, and interquartile ranges were employed for the description of quantitative variables. Absolute (*N*) and relative (%) frequencies were used for the description of qualitative variables. Student's *t*-test was used in order to compare quantitative variables among two groups. A variance analysis parametric test (ANOVA) was required for comparison of quantitative variables among three or more different groups. Levels of significance were two tailed and statistical significance was set to 0.05. The SPSS 17.0 software was used for the statistical analysis. 

## 3. Results

### 3.1. Descriptive Results

2,163 students with an average age of 9.9 (±1.1) years comprised our sample. Most of the participants, 72%, lived in an urban area, 84.2% of the participants were Greeks, whereas 15.8% had a different nationality, especially Albanian and Bulgarian, with skin type II instead of skin type III or IV for Greek students.

As far as the individual characteristics (as described in Table 1) were concerned, 16% of the participants belonged to the high-risk group. The high-risk group consisted of students' with at least four out of five of the following characteristics: fair skin colour, fair eyes colour, fair hair feckles, and moles. Interestingly enough, 53.8% of the students said they had fair complexion and, when exposed to the sun, their skin tended to get reddish and 68.1% had moles on their face or skin.

Behavioural patterns, habits, and attitudes regarding sun-protection measures were examined by 11 questions. 40.6% of the students said they always put a hat on when exposed to the sun, 52.6% said they wore hats sometimes but 6.8% of them said that they never wear hats. Only 36.3% said they always put on sunglasses when under the sun, 45.3% sometimes do, and 18.3% answered that they never put on sunglasses. 72.1% of the participants said they always apply sunscreen, 34.5% did not know the SPF, and only 46.3% said they preferred to stay in the shade when at the beach. 32.5% of the students said they like to be tanned, and 64.0% said they went to the beach more than 40 times last summer. Sunburn incidence rate (at least one sunburn during last summer) was an important finding in our study, since it reached 33.1%. 

### 3.2. Statistical Results

#### 3.2.1. Correlation of Students' Attitudes with Demographics

**Figure 1:**
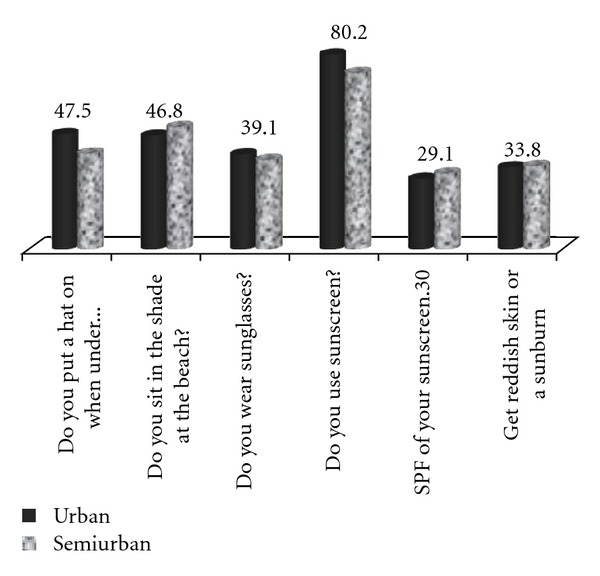
Students' behaviour according to living area.

Figure 1 and Table 2 illustrate attitudes of the participants according to area of residence and distance from the beach. There was a significant variation of attitudes regarding sun-protection measures according to how far from the beach the pupils lived. Another difference was noticed among those living in urban and those living in semiurban areas: more specifically, students living in urban areas had in general a better attitude regarding sun protection compared to students living in semiurban areas as it is shown in Figure 1.

#### 3.2.2. Correlation of Students' Attitudes with Age and Gender

**Figure 2:**
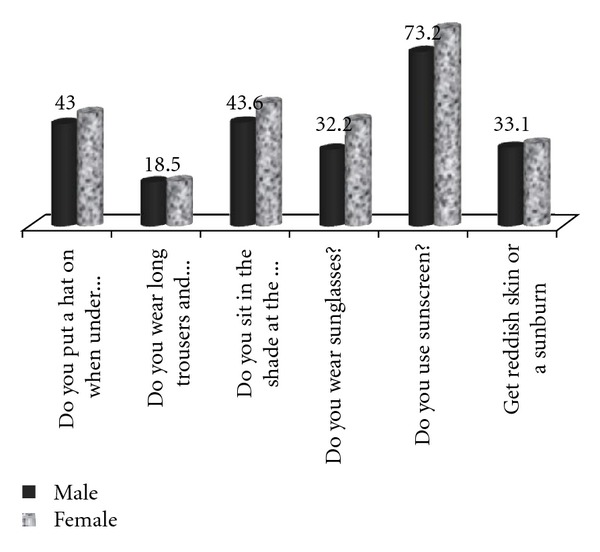
Students' behaviour according to gender.

Table 3 illustrates attitudes and behavioural patterns according to the students' age. There was a statistically significant difference in the students' attitudes regarding the sun according to their age. More specifically, higher age goes along with worse attitudes. Students up to 9 years old have better attitudes regarding hats, appropriate clothing, and sunscreen (*P* < 0.001). On the other hand, students aged 10 or older can apply sunscreen correctly and are aware of the right SPF (*P* < 0.001). Higher age means more visits to the beach last summer (*P* < 0.001), and higher sunburn incidence too (*P* < 0.001) (Figure 2). As far as gender was concerned, females had had in general a more cautious behaviour regarding sun exposure.

#### 3.2.3. Correlating Students' Attitudes with Nationality and Individual Characteristics (High-Risk Group)

Table 4 illustrates the students' attitudes and behaviours according to nationality and individual characteristics (high-risk group). Greek students had had a better sun-related behaviour compared to their non-Greek costudents. Greek students said they always wear a hat (46.4% versus 37.5%) and sit in the shade (49.7% versus 34.1%) compared to students of non-Greek nationality respectively. Those students who did not belong to the high-risk group had had a better attitude against sun protection in addition to those who belong to the high-risk group. They answered that they always wear a hat (46% versus 41.9%), they always use sunscreen (78.3% versus 77.5%) and the spf factor of the used sunscreen was >30 (30.2 versus 27.2%). On the other hand, only 27.2% of those who did belong to that high risk group (compared to 30.2% of the rest) used a sunscreen with SPF >30. Sunburn incidence was also significantly different among the two groups (31.9% versus 44.8%, *P* < 0.001).

#### 3.2.4. Correlating Sunburn Incidence with Frequency of Sun-Protection Measures

After correlating sunburn incidence at the last summer, with how often the students use protective measures, it was found that the less they use those measures, the higher the sunburn incidence rates. A mere 26.5% of those who reported having suffered a sunburn said they always wear a hat (*P* < 0.001), 39.4% sit in the shade (*P* = 0.016), 28.2% wear sunglasses (*P* = 0.045), and 31.3% like to be tanned (*P* < 0.001). 58.5% of those who said they always use sunscreen also said they had suffered a sunburn (*P* < 0.001), and 51.7% of those who used a sunscreen with SPF >30 also had suffered a sunburn. It is alarming that 83.2% of those who had suffered from a sunburn said they did not take any protective measures whatsoever (*P* < 0.001). In addition, those who reported that they had not suffered any sunburn the last summer said they always wear a hat 66.9%, always sit in the shade 61.9%, wear sunglasses 66.6%, and never use sunscreen 60.9%.

After multivariable regression analysis, we found that students who are at the beach usually have protective attitudes (mean 2.88, SD 0.83, *P* (*t*-test) <0,001) but in school they do not (mean 2.88, SD 0.84, *P* (*t*-test) = 0,117). Also, they usually refer that they used to stay under shadow (mean 2.88, SD 0.84, *P* = 0,017, Student's *t*-test).

## 4. Discussion

Because people receive 50 to 80 percent of their lifetime ultraviolet exposure by the age of 18, children are the most vulnerable. Recent studies point out that excessive exposure to the sun during childhood and adolescence, accumulation of solar radiation over the years, plenty episodes of severe sunburn, a large number of moles, a fair skin with blond or red hair and light-coloured eyes, living closer to the earth's equator, where the sun's rays are more direct, and a family history of melanoma are some important risk factors for developing various problems like skin damages (erythema, premature aging, skin cancer, and melanoma), eye damages (pterygium, cataract), and inhibition of the immune system [7, 12, 13]. 

Protecting children from the sun is a national priority for many countries. Accordingly, many education programmes have been designed in order to be implemented in schools [17, 20, 21, 27, 28, 31]. School environment is a potentially effective place to implement such intervention programmes, but in Greece many things remain to be done [21, 25].

In Greece, there is a lack of well-documented data regarding attitudes of 8–12 years old students about UV rays, possible risks, and sun-protection measures. A recent study, which took place in the same area and focused on teenagers, has recorded in detail knowledge levels and attitudes of that particular age group [21, 25]. The results of this study show that knowledge about melanoma and UVR is very poor among teenagers in the area studied.More specifically, 50% said that they wore a hat and stayed in the shade. Furthermore, only 50% of the participants reported using sunscreen with sufficient sun-protection factor, regarding sunburn incidence, 55.6% had had at least one sunburn last summer, while 17.3% had experienced sunburn with blisters, percentage much higher than the one of the present study, 33,1%. Knowledge regarding sun exposure of the Greek adolescents was insufficient and they reported more risky behaviours during summer months than primary students of the present study [25, 26]. There is an urgent need to portray the actual picture, in order for a comprehensive health education programme to be implemented. 

According to our results, most of the students (72%) live in a coastal, urban area; consequently, they go to the beach frequently each summer and spend a lot of time exposed to the sun,which is an important factor since these children are exposed to the sun much more than the rest, especially in the summer. On the other hand, those students who lived in semiurban areas (28%) are also exposed for too long to the sun, mostly because there are many open areas for them to gather and play for many hours. Other similar studies have investigated attitudes of children of similar age mainly from Australia and New Zealand, two countries surrounded by sea and have high melanoma incidence especially in younger ages [25, 31–36]. 

The amount of solar UV radiation reaching the Earth's surface is affected by several geographical, climatic, and meteorological factors including altitude, latitude, season, aerosols, thickness and distribution of clouds, stratospheric ozone, surface reflection, and time of day, with the solar zenith angle being the dominant factor of influence followed by clouds, aerosols, and stratospheric ozone. Apart from the tropics, the highest levels occur when the sun is at its maximum elevation, at around midday (solar noon) during the summer months. The closer to equatorial regions, the higher the UV radiation levels. The racial and ethnic differences in skin cancer rates are mostly due to skin colour and skin type, which is determined by the amount of melanin produced by specialized skin cells called melanocytes. Greece, due to its geographical location and the specific characteristics of the native population, remains in a little danger but our astonishing rates of sunburn, as it seems from our results, should not keep us from acting today together with other countries like Australia and New Zealand [7, 9, 16, 33].

One should not forget that the Greek society has become a multicultural society, and nonnative nationalities, like immigrants with skin type I or II from other countries like Albania and Bulgaria, who live for a long distance in Greece, may be at greater risk because of sun exposure. There are many studies focusing on this particular point, because globalization has given birth to many multicultural societies. The present study showed that students of non-Greek nationality fall short of sun-protection measures, which may be attributed to lower educational levels or cultural differences [37, 38]. 

**Figure 3:**
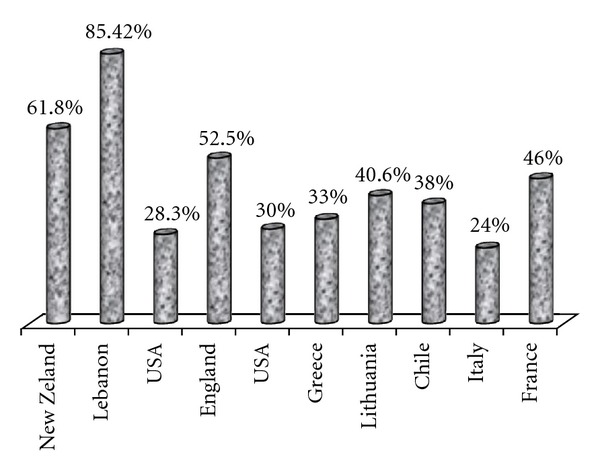
Sunburn incidence according to country.

According to the present study, there was an alarmingly high incidence of sunburns last summer, reaching 33.1% among the participating students. It was also found that students who did not take serious protective measures had a higher sunburn incidence. Also, sunscreen use does not seem enough to protect from sunburns because more than the half of the participants, who always used a sunscreen, reported at least one sunburn last summer. It is helpful here to point out the importance of the right use of sunscreen that children have to be taught. Several studies, Figure 3, from other countries have reported similar or different, but in the majority, disappointing results [37–45]. On the other hand, some recent studies mainly from Australia, New Zealand, and the United States have shown encouraging results and more specifically lower incidence of sunburns because of intensive educational programmes targeted at different age groups, but mainly at children and teenagers [46–49]. 

Correlation between attitudes and gender showed that females adopted a more cautious attitude compared to males, a common finding in several international studies too [45, 48, 50]. Mainstream culture regarding female appearance and the fact that females are in general more like to take care their body and skin, are perhaps the most common reasons for such attitudes [41–45, 50–52]. 

Older age, especially in teenagers, goes along with adopting a more cautious attitude against sun exposure, which has also been documented by other international studies as well. Maturing (both educationally and socially) is an important factor for adopting safer attitudes [20, 23, 31, 51, 52]. 

An alarming finding of the present study was the fact that the high-risk group had adopted riskier attitudes, although they should be much more cautious; future interventions and educational programmes should certainly focus on this particular point [38, 45, 48]. 

## 5. Conclusions

Health education programmes that have been implemented in Greece so far do not include sun-protection measures. In fact, correct and sufficient information regarding sun-protection measures for children and adolescents has not been included in the educational system yet. 

Educational psychology argues that knowledge growth and continuous education according to age can contribute immensely to attitude change in children and teenagers alike. Children of this age (8–12 year olds) are not expected to change directly their attitudes, since such a thing requires constant information and also a systematic implementation of intervention programmes both at school and at home. It has been noted that erroneous attitudes are not only unwise but dangerous as well. Adding the prolonged exposure because of the geographic position of the specific area, and also the total time that pupils remain under the sun even at school, it becomes obvious that interventions are simply necessary in order for sun-protection attitudes to be changed. 

Accumulation of solar radiation during childhood combined with sunburns might increase the possibility for developing melanoma or other skin cancer types later in life. Adopting sun-protection measures should be a focal point in designing and implementing educational programmes at schools. 

Educational approaches to sun safety in primary school may be effective for improving children's sun safety. Sun-protection policies in schools must be developed in consultation with the school community and the family too. Brief, standardized sun-protection education can be efficiently interwoven with school health education and can result in improved sun-protection behaviours. Schools should support the regular use of sunscreen, should seek to maintain and/or increase shaded spaces in the schoolyards, support the use of sun-protective clothing, hats, and sunglasses. Students should be encouraged to use the school's designated shaded areas, to wear hats and sun protective clothes, and to apply sunscreen themselves. Parents should be encouraged to supply 30+ broad-spectrum and water-resistant sunscreen as part of their child's school equipment and they must be informed of the school's sun-protection policy. This may be achieved by publishing the guidelines in the school handbooks and school newsletters.

When aiming at sun-protection and skin cancer prevention programmes, focusing on attitudes towards sun exposure and suntan may prove beneficial in terms of influencing sun-related behaviours.

## Figures and Tables

**Table 1:** Individual characteristics of the students.

	*N*	%
Total	**2163**	**100**

Male	1070	49.5
Female	1092	50.5

Nationality		
Greek	1898	87.8
Other	264	12.2

Distance of living from sea		
0–5 km	1576	72,9
10–15 km	88	4,1
15–20 km	155	7,2
>20 km	344	15,9

You are		
moles-skinned and rarely get sunburns	999	46.2
light-skinned and easily get sunburns	1163	53.8

Your eye colour is		
dark	1625	75.2
light	537	24.8

Your hair colour is		
dark	1483	68.6
light	679	31.4

Do your have freckles on your face/body		
No	1634	75.6
Yes	528	24.4

Do you have any moles on your face/body		
No	690	31.9
Yes	1472	68.1

High-risk group*		
No	1816	84
Yes	346	16

*The high-risk group consisted of students' with at least four out of five of the following characteristics: fair skin colour, fair eyes colour, fair hair freckles, and moles.

**Table 2:** Students' behaviour according to place of residence.

		Distance from the sea				
		0–5 km	10–15 km	15–20 km	>20 km	*P* Pearson's *x* ^²^ test
		*N* (%)	*N* (%)	*N* (%)	*N* (%)	
Do you put a hat on when under the sun?	Always	748 (47.5)	48 (54.5)	21 (13.5)	163 (47.4)	**<0.001**
Do you sit in the shade at the beach?	Always	737 (46.8)	44 (50)	64 (41.3)	188 (54.7)	**0.019**
Do you wear sunglasses?	Always	616 (39.1)	51 (58)	23 (14.8)	142 (41.4)	**<0.001**
Do you use sunscreen?	Always	1263 (80.2)	61 (69.3)	102 (65.8)	264 (76.7)	**<0.001**
What was the SPF of your sunscreen?	Over 30	457 (29.1)	21 (23.9)	23 (14.8)	140 (40.7)	**<0.001**
Do you like to be tanned?	No	920 (58.4)	55 (62.5)	90 (58.1)	177 (51.5)	**<0.001**
	No	921 (58.5)	65 (73.9)	68 (43.9)	202 (58.7)	
Last summer, did you get reddish skin or a sunburn on your face or body?	Yes	532 (33.8)	19 (21.6)	75 (48.4)	108 (31.4)	**<0.001**
	I don't remember	122 (7.7)	4 (4.5)	12 (7.7)	34 (9.9)	
	Less than 20	125 (8.4)	7 (8)	9 (5.8)	56 (16.3)	
How many times did you go to the beach (for a swim) last summer?	20 to 40	173 (11.7)	18 (20.5)	28 (18.1)	63 (18.3)	**<0.001**
	Over 40	1067 (72.1)	58 (65.9)	97 (62.6)	180 (52.3)	
	I don't remember	115 (7.8)	5 (5.7)	21 (13.5)	45 (13.1)	

**Table 3:** Students' behaviour according to age and gender.

			Age		
		<9	9-10	>10	*P* Pearson's *x* ^²^ test
		*N* (%)	*N* (%)	*N* (%)	
Do you put a hat on when under the sun?	Always	178 (51.6)	679 (46.7)	123 (33.9)	**<0.001**
Do you wear long trousers and long-sleeve shirts when under the sun?	Always	92 (26.7)	270 (18.6)	42 (11.6)	**<0.001**
Do you sit in the shade at the beach?	Always	173 (50.1)	693 (47.7)	167 (46)	0.538
Do you wear sunglasses?	Always	142 (41.2)	549 (37.8)	141 (38.8)	0.119
Do you use a sunscreen?	Always	295 (85.5)	1131 (77.8)	264 (72.7)	**0.001**
Last summer, did you get reddish skin or a sunburn on your face or body?	No	226 (65.5)	826 (56.8)	204 (56.2)	**0.002**
	Yes	89 (25.8)	505 (34.7)	140 (38.6)	

**Table 4:** Students' behaviour according to nationality and risk group.

		Nationality			High-risk group*		
		Greek	Other	*P* Pearson's *x* ^²^ test	No	Yes	*P* Pearson's *x* ^²^ test
		*N* (%)	*N* (%)		*N* (%)	*N* (%)	
Do you put a hat on when under the sun?	Always	881 (46.4)	99 (37.5)	**0.023**	835 (46)	145 (41.9)	**0.049**
Do you sit in the shade at the beach?	Always	943 (49.7)	90 (34.1)	**<0.001**	857 (47.2)	176 (50.9)	0.210
	Sometimes/Never	955 (50.3)	174 (65.9)		959 (52.8)	170 (49.1)	
Do you wear sunglasses?	Always	723 (38.1)	109 (41.3)	0.459	699 (38.5)	133 (38.4)	0.905
Do you use a sunscreen?	Always	1515 (79.8)	175 (66.3)	**<0.001**	1422 (78.3)	268 (77.5)	0.633
What was the SPF of your sunscreen?	Over 30	587 (31)	54 (20.5)	**0.001**	547 (30.2)	94 (27.2)	0.522
	No	1116 (58.8)	140 (53)		1097 (60.4)	159 (46)	
Last summer, did you get reddish skin or a sunburn on your face or body?	Yes	646 (34)	88 (33.3)	**0.001**	579 (31.9)	155 (44.8)	**<0.001**
	I don't remember	136 (7.2)	36 (13.6)		140 (7.7)	32 (9.2)	
How many times did you go to the beach (for a swim) last summer?	Less than 20	158 (8.8)	39 (14.8)	**<0.001**	153 (8.8)	44 (13.6)	**0.027**
	Over 40	1250 (69.3)	152 (57.6)		1201 (68.9)	201 (62.2)	

*The high-risk group consisted of students' with at least four out of five of the following characteristics: fair skin colour, fair eyes colour, fair hair freckles, and moles.
